# Diagnostic Aspects and Management Strategies in Primary and Metastatic Intestinal Melanoma: A Literature Review

**DOI:** 10.3390/medsci14020281

**Published:** 2026-05-31

**Authors:** Alexandra Caziuc, Radu Alexandru Ilieș, George Ionuț Golea, Andrada Larisa Deac, George Călin Dindelegan

**Affiliations:** 1 Department of General Surgery, “Iuliu Hațieganu” University of Medicine and Pharmacy, 400012 Cluj-Napoca, Romania; caziuc.alexandra@umfcluj.ro (A.C.); george.dindelegan@umfcluj.ro (G.C.D.); 2 First Surgical Unit, Emergency County Hospital Cluj, 400006 Cluj-Napoca, Romania; 3 Faculty of Medicine, “Iuliu Hațieganu” University of Medicine and Pharmacy, 400012 Cluj-Napoca, Romania; ilies.radu.alexandru@elearn.umfcluj.ro; 4 Department of Pharmacology, Toxicology and Clinical Pharmacology, “Iuliu Hațieganu” University of Medicine and Pharmacy, 400012 Cluj-Napoca, Romania

**Keywords:** primary small bowel melanoma, metastatic intestinal melanoma, differential diagnosis, immunohistochemistry, surgery

## Abstract

**Background/Objectives**: Intestinal malignant melanoma is a rare entity, most commonly presenting as metastatic disease from a cutaneous primary source. The distinction between primary and secondary intestinal melanoma remains challenging, yet it has important diagnostic, therapeutic, and prognostic implications. This study aims to highlight the diagnostic difficulties and therapeutic considerations associated with intestinal melanoma. **Methods**: A narrative literature review was conducted using the PubMed database, only including articles published between January 2015 and December 2025. Case reports, case series, and reviews that described primary-like (i.e., presumed primary) or metastatic small bowel melanoma were considered eligible. Extracted data consisted of clinical presentation, diagnostic workup, histopathological and immunohistochemical features, treatment strategies, and outcomes. **Results**: Twenty articles met the inclusion criteria, comprising ten reporting primary intestinal melanoma and ten reporting metastatic intestinal melanoma. Primary-like intestinal melanoma was frequently solitary, amelanotic, and occurred in patients without a prior history of melanoma, whereas metastatic disease was usually multifocal and associated with a known cutaneous primary source. Clinical manifestations were nonspecific, most frequently including anemia, gastrointestinal bleeding, abdominal pain, or intestinal obstruction. Immunohistochemistry confirmed melanocytic origin in each case, but could not reliably differentiate primary from metastatic disease. Surgical resection remained the cornerstone of treatment, with systemic therapy reserved primarily for metastatic cases. **Conclusions**: Diagnosis of primary intestinal melanoma relies on excluding other primary sites through comprehensive clinical and imaging evaluations. Early detection using advanced endoscopic techniques and multidisciplinary management are vital for optimizing outcomes. While metastatic intestinal melanoma carries a poor prognosis, complete surgical resection of primary lesions has been associated with improved outcomes in selected patients.

## 1. Introduction

Malignant melanoma accounts for approximately 1–3% of all gastrointestinal malignancies. Most of these are secondary lesions rather than primary tumors. However, both primary and metastatic intestinal melanomas exhibit greater biological aggressiveness and poorer prognosis than melanoma at other anatomical sites [1]. Even though only 0.8–4.7% of patients with this disease report clinically apparent gastrointestinal metastases from melanoma, postmortem studies demonstrate gastrointestinal involvement in up to 60% of patients with disseminated disease, emphasizing the frequently occult nature of intestinal metastases [2]. Early-stage asymptomatic metastatic disease is rarely documented, as only 1–5% of cases are diagnosed incidentally on imaging examinations (these cases being associated with improved therapeutic outcomes) [1].

Gastrointestinal metastases that arise from melanoma (with an unknown primary cutaneous origin) account for approximately 4–9% of all cases, and despite advances in diagnostic methods, only 1.5–4.4% of gastrointestinal metastases are identified during the patient’s lifetime [3].

Melanoma can originate at any site within the gastrointestinal mucosa, but it most commonly arises in the anorectal region, particularly the anal canal (31.4%) and rectum (22.2%), followed by the oropharyngeal region (32.8%). Less frequently involved sites include the esophagus (5.9%), stomach (2.7%), small intestine (2.3%), gallbladder (1.4%), and large intestine (0.9%) [4].

This work aims to explore the differential diagnosis between primary and metastatic intestinal melanoma and to analyze the therapeutic strategies and prognostic implications, illustrating the importance of multidisciplinary decision-making and modern systemic therapies for this disease.

## 2. Materials and Methods

Considering that intestinal malignant melanoma is a rare pathology with limited evidence, mostly consisting of case reports and small case series, the study design of a narrative review was chosen as the most appropriate approach.

**Figure 1:**
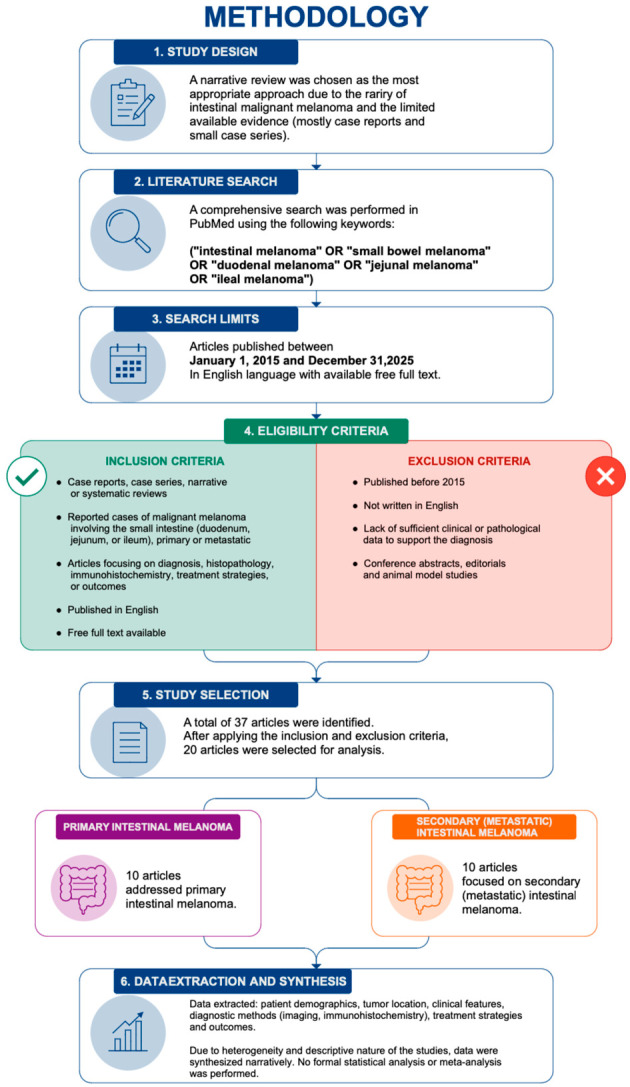
Flowchart of the study methodology. Schematic representation of the study workflow, illustrating the sequential stages from study design and literature search to eligibility assessment, study selection, and data synthesis. The diagram highlights the screening process, the division of included studies into primary and secondary intestinal melanoma, and the final step of narrative data integration.

A search in the literature was performed in PubMed to identify articles on the topic of intestinal malignant melanoma (Figure 1). This review was not conducted according to PRISMA guidelines, which represents a limitation inherent to its narrative design. The following search strategy was used: (“intestinal melanoma” OR “small bowel melanoma” OR “duodenal melanoma” OR “jejunal melanoma” OR “ileal melanoma”). The search was restricted only to articles that were published between 1 January 2015 and 31 December 2025, with the intention of including studies with modern diagnostic and therapeutic approaches.

Subsequently, we defined eligibility criteria for narrative synthesis. Included articles consisted of case reports, case series, and narrative or systematic reviews. Some publications had a hybrid structure, combining review components with original clinical cases. Only articles published in English were considered for the review. The availability of full-text articles was required to allow for the complete extraction of clinical and histopathological data for narrative synthesis. Studies were considered eligible if they reported cases of malignant melanoma involving the small intestine (either duodenum, jejunum, or ileum), regardless of whether the lesions were considered primary or metastatic. We included articles focusing on the process of diagnosis, histopathological characteristics, immunohistochemistry, treatment strategies, or outcomes. Articles were excluded if they were published before 2015, if they were written in any language other than English, or if they lacked sufficient clinical or pathological data to further support the diagnosis of intestinal malignant melanoma. Abstracts from conferences, editorials, and animal model studies were also excluded.

From a total number of 37 articles identified through the literature search, 20 met the aforementioned criteria and were selected for analysis. For the purpose of analysis, included cases were: (1) intestinal melanoma presenting without an identifiable primary lesion (primary-like/melanoma of unknown primary spectrum) and (2) intestinal involvement in patients with known melanoma or disseminated disease (metastatic intestinal melanoma). It is acknowledged that cases in the first category might represent a heterogeneous group, including true primary intestinal melanoma and melanoma of unknown primary origin. This grouping was performed for analytical purposes only and does not imply definitive diagnostic reclassification of individual cases.

Data on patient demographics, location of the tumor, clinical features, diagnostic methodology (including imaging and immunohistochemistry), treatment strategies, and outcomes were extracted from each eligible study. Following this, the extracted information was synthesized narratively due to the heterogeneity and descriptive nature of the available reports. No formal statistical analysis or meta-analysis was feasible, given the rarity of this disease.

## 3. Results

### 3.1. Overview of the Included Studies

Twenty articles were included in our analysis. Ten reports described intestinal melanoma without an identifiable primary lesion, representing a heterogeneous group including true primary intestinal melanoma and melanoma of unknown primary (MUP)-spectrum cases. These cases included both solitary intestinal lesions and multifocal presentations without documented extraintestinal primary tumors. The remaining ten reports described intestinal involvement in patients with known cutaneous or systemic melanoma or with clear evidence of disseminated disease, consistent with metastatic intestinal melanoma.

Gómez-Uranga et al. (2022) presented in their work the case of a 77-year-old woman who presented with anemia [5]. Capsule endoscopy was performed and revealed an ulcerated lesion located in the distal jejunum and proximal ileum. Analysis of the surgical resection specimen confirmed amelanotic melanoma with S100 positivity, and no other primary tumor was identified. The patient remained disease-free for three years, indicating a likely primary intestinal melanoma according to Sachs criteria [5].

Eng et al. (2021) presented the case of a 61-year-old male patient with intermittent abdominal distension and symptomatic anemia [6]. Imaging suggested small bowel intussusception with an omental mass. Consequently, the patient underwent exploratory laparotomy and segmental bowel resection, and histopathological examination confirmed melanoma involving the gastrointestinal tract; the authors reported it as primary gastrointestinal melanoma. The surgical intervention was early and allowed for histologic diagnosis; it prevented complete obstruction and guided further treatment. Also, the authors classified the lesion as primary gastrointestinal melanoma based on the exclusion of an identifiable extraintestinal primary site, while acknowledging the diagnostic uncertainty due to the presence of multiple intestinal lesions and the possibility of an occult or regressed cutaneous primary melanoma [6].

Mattit et al. (2024) described the case of a 57-year-old man who presented with chronic constipation, abdominal pain and distension [7]. CT imaging revealed jejunal wall thickening, but endoscopy and biopsy were inconclusive. Intraoperatively, a segmental enterectomy was performed, and pathology confirmed the diagnosis of melanoma. No primary cutaneous melanoma was found on further examination, suggesting a primary small bowel melanoma. The authors indicate that this disease is rare, usually asymptomatic, and can present with obstruction. Early diagnosis and surgical management are important for symptom control and prognosis [7].

Spiridakis et al. (2015) reported the case of a 68-year-old Caucasian male patient who presented with weakness, rectorrhagia and weight loss [8]. Endoscopy revealed an ulcerated jejunal mass, and consequently, an exploratory laparotomy with small bowel resection was performed, confirming the presence of a tumoral mass involving the full thickness of the bowel. Immunohistochemistry was positive for HMB-45, Melan-A, and S100. Extensive work-up, including skin, ocular, gastrointestinal, chest, brain, and liver, together with bone imaging, found no primary cutaneous or other sites of melanoma. At 11 months of follow-up, no metastases were found, supporting the diagnosis of primary small bowel melanoma [8].

Ait Idir et al. (2016) reported, in their work, the case of a 75-year-old woman who presented with gastrointestinal bleeding and was later found to have a small bowel melanoma [9]. Diagnosis was established only after segmental intestinal resection, based on histological and immunohistochemical findings. Extensive postoperative evaluation failed to identify any cutaneous, ocular, or other gastrointestinal primary lesion, further supporting the diagnosis of primary small intestine melanoma (even though the possibility of an undetected regressed primary melanoma could not be completely excluded) [9].

Kilambi et al. (2017) detailed the case of a 35-year-old man who presented with weight loss and abdominal pain [10]. Imaging and endoscopy emphasized a large duodenal mass that involved the superior mesenteric vessels, with biopsy confirming malignant melanoma. Clinical examination and a whole-body PET-CT excluded other primary sites. Next, the patient underwent surgery (pancreaticoduodenectomy with resection of the superior mesenteric vein and segmental colectomy), followed by adjuvant treatment with temozolomide. Even if the patient developed hepatic metastases at 6 and 10 months (which were successfully treated via radiofrequency ablation), he was stable at 32 months follow-up, illustrating the benefit of a multimodal approach [10].

Hadjinicolaou et al. (2016) reported the case of small intestine melanoma in a 60-year-old male patient presenting with melena and anemia, in whom conventional endoscopic and radiologic investigations did not reveal any abnormalities [11]. However, capsule endoscopy identified a jejunal mass, which was surgically resected and confirmed as melanoma after histopathological report. Extensive examination did not find any evidence of a cutaneous primary lesion. The authors debated whether small bowel melanomas represent true primary tumors or metastases from occult or regressed cutaneous melanoma [11].

Shin et al. (2017) described a case of primary small bowel melanoma which occurred in a 74-year-old man presenting with recurrent melena [12]. Both upper and lower endoscopic evaluations were normal. However, abdominal CT revealed an ileal mass with ileo-ileal intussusception. Via single-balloon enteroscopy, direct visualization and biopsy of the lesion were possible, confirming the diagnosis of melanoma. Clinical examination revealed no evidence of a cutaneous primary source. The authors acknowledge the diagnostic value of balloon-assisted enteroscopy in cases of obscure gastrointestinal bleeding and also claimed it to be the first reported case of primary small bowel melanoma in South Korea [12].

Olatoke et al. (2019) described in their work a case of jejunal melanoma presenting as intussusception [13]. It occurred in a 63-year-old female patient with abdominal pain, weight loss, and intestinal obstruction. The jejunal tumor represented the lead point for intussusception and was later confirmed as melanoma after histopathological report. Even though no cutaneous lesions were identified, the absence of PET imaging could not exclude any extra-intestinal primary tumor that was either occult or regressed. In their literature review, the authors supported that most gastrointestinal melanomas are secondary tumors and highlighted their diagnostic difficulty posed by nonspecific symptoms [13].

Kouladouros et al. (2015) reported a case of primary intestinal melanoma that presented with recurrent intussusception in a 42-year-old female patient (with no prior history of melanoma) [14]. Imaging emphasized ileocolic intussusception and, consequently, the patient underwent surgical exploration, which identified multiple pigmented tumors in the small bowel. Histopathological exam confirmed intestinal melanoma, while clinical, endoscopic, and PET-CT excluded any other sites of primary lesions. Because of the multifocal intestinal involvement, curative resection was not feasible, and only limited palliative surgery was performed to relieve the obstruction [14].

Sinagra and Sciumè (2020) reported the case of a 64-year-old patient with acute abdominal pain, vomiting, and bowel obstruction [15]. Imaging and emergency laparotomy showed a bulky ileal tumor, which caused small bowel obstruction [15]. A total of 20 cm of ileum, along with mesentery and lymph nodes, were resected and showed a primary epithelioid malignant melanoma, with S100 and Melan-A positivity; three out of seven lymph nodes were metastatic. The patient completed adjuvant chemotherapy with dacarbazine and continued regular oncological surveillance. The authors emphasize that even if primary small bowel melanoma is extremely rare, it ought to be considered in some cases of unexplained bowel obstruction [15]. Although the authors originally reported this lesion as a primary epithelioid malignant melanoma of the small bowel, according to the classification in the Discussion (Section 4.3), this case does not fulfill the strict criteria for primary intestinal melanoma due to the presence of regional lymph node involvement and the detection of an additional PET-positive extraintestinal lesion. Even if no primary cutaneous, ocular, or mucosal melanoma was identified, the case is most appropriately classified as intestinal melanoma of unknown primary origin, reflecting either a regressed primary lesion or an undetected extraintestinal primary.

Fernandez Noël et al. (2023) reported a 68-year-old man with a history of cutaneous melanoma treated in 2020 who presented with melena in 2022 [16]. CT scan showed focal nodular thickening of a small bowel loop, and capsule endoscopy identified a 10 mm excrescent lesion in the mid-jejunum with active bleeding. PET-CT confirmed hypermetabolic mesenteric adenopathies and duodenal/jejunal involvement, consistent with metastatic melanoma. The patient underwent intestinal resection (including the lesions and palpable lymph nodes), and pathology further confirmed intestinal melanoma metastases. The authors highlight that capsule endoscopy improves the detection of small bowel lesions not visible on conventional endoscopy, allowing for better preoperative planning [16].

Aryan et al. (2023) reported a series of three patients with ages ranging from 55 to 81 years with a history of cutaneous melanoma who presented with abdominal discomfort and anemia; one also presented with upper GI bleeding (melena) [17]. CT and PET scans revealed multiple hypermetabolic small bowel lesions. Double-balloon enteroscopy identified a few black, pedunculated, and sessile jejunal masses, some of them being ulcerated and bleeding. Biopsies confirmed melanoma with positive staining for S100, SOX-10, and Melan-A/MART-1. All three patients were referred for oncology care; one of them died two months after diagnosis, while the other two were managed at external institutions [17].

Zoumpos et al. (2019) reported in their work the case of a 67-year-old man with a history of malignant melanoma who presented with anemia and vertigo [18]. Standard GI bleeding work-up, including gastroscopy, colonoscopy, abdominal ultrasound, and CT scan; none of them were able to identify the source. However, video capsule endoscopy revealed numerous hemorrhagic small bowel metastases. Later, diffuse metastatic disease was discovered (involving the heart, brain, liver, spleen and bone) and the case was further managed conservatively/palliatively [18].

Galindo et al. (2023) discussed a case that was initially diagnosed as primary small bowel melanoma presenting with obstruction and hemorrhage [19]. Subsequent work-up revealed a small cutaneous melanoma of the left thigh, showing that the small bowel lesion was in fact metastatic. The authors underscore the importance of investigating all presumed primary small bowel melanomas for an alternative primary lesion and advise reconsidering the classification of such cases as primary small bowel melanoma only when no cutaneous source is immediately identified [19].

Drober et al. (2025) discussed the case of an 81-year-old male patient with a history of spindle cell melanoma of the scalp (who had been treated five years earlier) and who presented with severe anemia [20]. Rectal examination revealed melena, while upper and lower endoscopy failed to identify any source of bleeding. CT scan and enteroscopy emphasized an ulcerated jejunal mass occupying more than 50% of the lumen. The patient underwent laparoscopic segmental resection of ten centimeters of jejunum, and histopathological analysis confirmed metastatic malignant melanoma. The authors emphasize that small bowel metastases should be considered in patients with a history of melanoma presenting with unexplained anemia or gastrointestinal bleeding [20].

Bakula et al. (2024) described the case of a 41-year-old woman with a history of cutaneous melanoma, which had been excised five years earlier, who presented with acute small bowel obstruction [21]. Imaging illustrated an enteroenteric intussusception, which was demonstrated surgically. Intraoperatively, a pigmented ileal mass acting as the lead point was identified. Later, it was histologically proven to be metastatic cutaneous melanoma. This case highlights the exceptional rarity of small bowel melanoma metastases causing intussusception [21].

Vigorita et al. (2015) discussed, in their work, the case of a 48-year-old woman who developed small bowel intussusception caused by metastatic lesions of melanoma 15 years after complete excision of the primary cutaneous tumor [22]. The patient underwent emergency surgical resection, with negative margins confirmed on histopathology. This case illustrates the nonspecific clinical presentation of intestinal melanoma metastases and shows the importance of considering late gastrointestinal recurrence in patients with a history of melanoma [22].

Wu et al. (2022) reported the case of a patient with no prior history of melanoma who presented with gastrointestinal bleeding and small bowel obstruction [3]. Imaging revealed active hemorrhage; hence, diagnostic laparoscopy and subsequent small bowel resection were performed. Histopathological analysis of the specimen confirmed jejunal metastatic melanoma, necessitating additional resections. This case underscores the diagnostic difficulty of gastrointestinal metastases of melanoma [3].

Gomez Pons et al. (2024) discussed the case of a 76-year-old female patient with primary anorectal mucosal melanoma who developed recurrent gastrointestinal bleeding and anemia caused by small bowel metastases (approximately 4.5 years following initial diagnosis) [23]. The patient was successfully treated via laparoscopic resection of the small intestine. This case highlights the tendency of mucosal melanoma to spread along the gastrointestinal tract, where it might cause complications such as bleeding or intussusception [23].

### 3.2. Integrated Analysis of the Included Literature

In the group of primary intestinal melanoma studies (*n* = 10), all authors established diagnosis based on the absence of any identifiable primary site following extensive work-up [5,6,7,8,9,10,11,12,13,14]. Explicit reference to diagnostic criteria relying on exclusion (including Sachs criteria) was made in four out of ten studies [5,8,11,12], while three studies acknowledged the possibility of an undetected or even regressed cutaneous primary lesion [9,11,13]. Clinically, anemia or gastrointestinal bleeding was reported in 6/10 cases [5,6,8,9,11,12], abdominal pain in 5/10 [6,7,10,13,14], and bowel obstruction or intussusception occurred in 3/10 [6,13,14]. Some authors (4/10) emphasized the nonspecific or chronic nature of symptoms contributing to delayed diagnosis [6,7,10,13].

From a diagnostic perspective, eight out of ten studies stated that preoperative investigations were inconclusive (or failed to establish a definitive diagnosis) [6,7,8,9,11,12,13,14], with six studies explicitly mentioning that diagnosis was established only after surgical resection and histopathological examination [7,8,9,10,13,14]. Capsule endoscopy was regarded as a useful diagnostic tool in three cases [5,11,13], while balloon-assisted enteroscopy was reported in a single study as allowing for direct visualization and biopsy [12]. Immunohistochemical confirmation was consistent across all cases (10/10), with S100 positivity reported in five studies [5,8,10,11,12] and Melan-A/HMB-45 expression in four studies [8,10,12,14]. All authors emphasized surgery as the cornerstone of management (10/10), serving a diagnostic role in 6/10 cases [7,8,9,10,13,14] and a potentially curative role in 5/10 [5,6,8,10,11]. Favorable outcomes (regarding disease-free survival after analysis) were reported in three cases [5,8,10], while most authors (7/10) explicitly emphasized the rarity of primary intestinal melanoma [7,9,10,11,12,13,14].

In the metastatic intestinal melanoma group (*n* = 10), a prior history of melanoma was documented in seven out of ten cases [16,17,18,20,21,22,23], while three cases had no known primary at presentation, with the diagnosis established subsequently [3,15,19]. The most frequent clinical manifestations were melena and anemia (7/10) [16,17,18,19,20,22,23], followed by bowel obstruction (5/10) [3,15,19,21,22] and intussusception (3/10) [21,22,23]. Active gastrointestinal bleeding was specifically reported in four studies [3,16,18,23]. Similar to primary cases, standard diagnostic investigations were initially negative in 5/10 cases [18,19,20,22,23], while capsule endoscopy contributed significantly to lesion detection in three studies [16,18,20]. PET-CT was highlighted as important for staging and detection of additional metastatic sites in three cases [16,17,19]. Histopathological confirmation was required in all cases (10/10), often supported by immunohistochemistry [3,15,16,17,18,19,20,21,22,23].

Regarding disease extent, four studies reported disseminated metastatic disease involving multiple organs [17,18,20,23], while lymph node involvement was documented in two cases [15,16]. Several authors (4/10) emphasized the potential for late gastrointestinal metastases, occurring years after the primary melanoma had been treated [20,21,22,23]. Surgical intervention was performed in the majority of cases (8/10) [3,15,16,20,21,22,23], mainly for complication management (obstruction, bleeding), rather than curative intent. On the whole, authors consistently highlighted the diagnostic difficulty, nonspecific presentation, and aggressive nature of metastatic intestinal melanoma, as well as the need for high clinical suspicion, particularly in patients with prior history of melanoma [17,18,19,20].

## 4. Discussion

### 4.1. Comparative Analysis of Primary and Secondary Intestinal Melanoma

The difference between primary and secondary intestinal melanoma remains one of the most debated aspects regarding gastrointestinal melanoma. Within this spectrum, melanoma of unknown primary remains a major confounding category that overlaps clinically and pathologically with presumed primary intestinal melanoma. Multiple authors stated that the majority of intestinal melanomas are of metastatic origin, usually arising years after prior treatment of a cutaneous primary melanoma [13,18,19].

Nonetheless, a small number of cases respect the strict diagnostic criteria for primary disease, which include the absence of any cutaneous, ocular, or other mucosal lesions after extensive clinical and imaging evaluation [5,8,10].

Primary small bowel melanomas tend to present as solitary lesions and are frequently amelanotic. They are diagnosed after a long period following their occurrence, due to nonspecific symptoms such as obscure gastrointestinal bleeding, anemia, abdominal pain, or impaired abdominal transit [7,11].

In contrast, metastatic intestinal melanoma often presents with multifocality, systemic spread, and poorer prognosis (compared to primary tumors). Usually, it requires surgical intervention for severe complications such as obstruction or intussusception [21,22].

Notably, several authors acknowledge that immunohistochemistry cannot properly differentiate primary from metastatic disease, justifying why thorough clinical examination and long-term follow-up are essential [9,19].

Recent advances in balloon-assisted enteroscopy and capsule endoscopy have significantly improved the detection rate of small bowel lesions and must be considered early in patients with unexplained anemia or bleeding with inconclusive imaging or basic endoscopic examinations [11,12,16].

**Figure 2:**
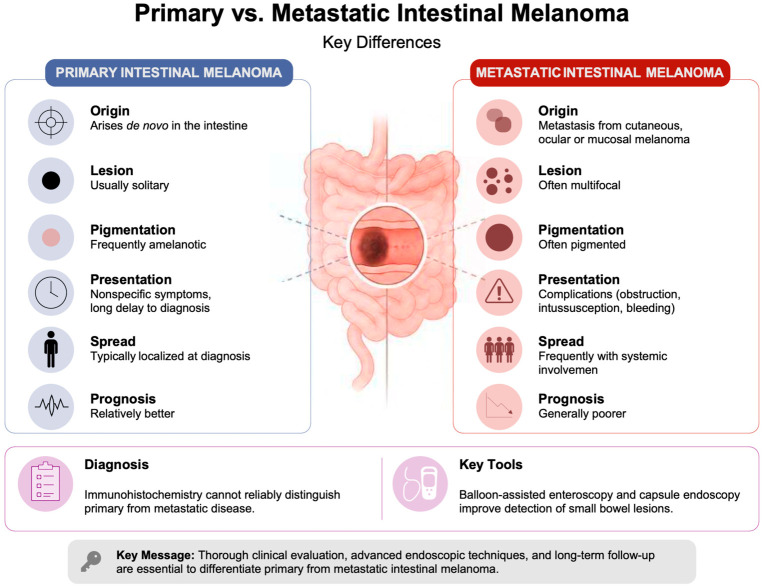
Summary of the main differences between primary and metastatic intestinal melanoma according to the reviewed literature.

A comparative analysis of primary versus metastatic intestinal melanoma is presented in Table 1 and Figure 2.

Systemic treatment strategies for intestinal melanoma remain highly heterogeneous and are mainly derived from the case reports included in the present review. In the analyzed literature, older therapeutic approaches included dacarbazine- or temozolomide-based chemotherapy, often used in metastatic or advanced disease settings [10,15]. More recently, multimodal approaches combining surgery with systemic therapy have been reported, particularly in metastatic cases with limited disease burden [10,17,20]. In selected patients, surgical resection of intestinal metastases was followed by oncological surveillance or adjuvant systemic treatment depending on disease stage and overall metastatic spread [15,16,20,21,22,23].

Instead of representing strictly distinct entities, primary and metastatic intestinal melanoma likely exist along a biological and diagnostic spectrum, including metastatic melanoma of known primary and other rare cases that fulfil the criteria for true primary intestinal melanoma. Several cases that are initially considered as primary gastrointestinal melanoma might ultimately represent metastatic disease from an occult or regressed primary lesion, as highlighted by reports in which an apparent primary intestinal lesion was later reclassified after the identification of a cutaneous primary site [13,18,19]. This highlights the significant diagnostic uncertainty inherent to this entity.

### 4.2. Systemic Therapy and Molecular Insights in Intestinal Melanoma

Across the included studies, the molecular profile of intestinal melanoma was variably reported, as most cases lacked comprehensive genomic profiling. Immunohistochemical confirmation of melanocytic origin was generally performed, with positivity for S100, Melan-A, HMB-45, and SOX-10; however, the aforementioned immunohistochemical markers confirm melanocytic lineage, but do not make it possible to differentiate between primary and metastatic intestinal melanoma [5,8,10,11,12,17,20].

Systematic molecular analysis concerning BRAF or KIT mutational status, as well as evaluation of PD-1/PD-L1 expression or tumor-infiltrating lymphocytes, was not reported in the included studies, limiting reliable estimations of mutation frequency in intestinal melanoma.

Despite these limitations, extrapolation from melanoma biology supports the promising role of immune checkpoint inhibitors for managing metastatic disease. In the reviewed cases reporting metastases, systemic therapy was overall reserved for disseminated disease or in postoperative adjuvant settings, while surgical resection remained the primary therapeutic approach for symptom control and local disease management [3,15,16,20,21,22,23]. In one reported case, the patient underwent adjuvant dacarbazine chemotherapy followed by oncological surveillance [15]. However, melanoma treatment paradigms suggest that immune checkpoint blockade might represent a rational therapeutic option in advanced intestinal melanoma. This hypothesis mainly derives from its established efficiency in metastatic melanoma in general (widely mentioned in the available literature for the management of advanced disease) [24,25,26,27,28].

### 4.3. Diagnostic Considerations and Clinicopathological Stratification of Intestinal Melanoma

Instead of relying solely on historical labels reported in individual publications, cases may be more appropriately interpreted according to objective clinicopathological features and patterns of disease distribution.

The diagnosis of primary intestinal melanoma remains one of exclusion and is supported by a set of proposed clinical criteria known as Sachs criteria [29,30]. These include:

(1) Identification of a biopsy-proven melanoma localized in a single intestinal site;
(2) No evidence of extraintestinal disease at the time of diagnosis following comprehensive staging;
(3) Disease-free survival period of minimally 12 months after initial treatment.

Together, these criteria have the objective to optimize diagnostic confidence; however, they do not completely exclude the possibility of an occult or regressed primary melanoma arising from cutaneous, ocular or other mucosal sites [29,30].

Cases with no identifiable primary lesion but not fully meeting proposed criteria for primary intestinal melanoma may be more appropriately categorized as intestinal melanoma of unknown primary origin, acknowledging the possibility of either regressed cutaneous melanoma or undetected primary tumor.

Additional clinicopathological characterization may also consider:

(1) The presence or absence of a known extraintestinal primary melanoma (cutaneous, ocular, oral, or anorectal);
(2) Tumor distribution pattern, distinguishing solitary intestinal/mesenteric lesions from multifocal intestinal or diffuse mesenteric involvement.

This dual-axis approach better reflects the biological spectrum of disease and reduces misclassification bias inherent to strict primary versus metastatic categorization.

Despite these diagnostic and stratification approaches, definitive differentiation between primary and metastatic intestinal melanoma remains challenging in many cases because of the possibility of occult or regressed primary lesions. Hence, any classification should be interpreted with caution and in the context of comprehensive clinical and radiological evaluation.

### 4.4. Limitations of the Current Study

The current review is subject to several limitations, mainly reflecting the rarity of intestinal malignant melanoma and the characteristics of the available evidence. Most of the included studies consist of case reports and small case series, which inherently limits the generalizability of the findings and increases the risk of publication bias, as unusual or complex cases are more likely to be reported. In addition, the heterogeneity of the available data poses a significant challenge, with considerable variability in clinical presentation, diagnostic work-up, and reporting standards across studies. Differences regarding the use and availability of imaging modalities, immunohistochemical markers, and molecular profiling further complicate direct comparisons.

The difficulty in accurately distinguishing between primary and metastatic intestinal melanoma represents another important limitation. Moreover, potential classification bias exists within the published literature itself, as many reported cases were categorized based primarily on the absence of an identifiable extraintestinal primary lesion rather than on standardized clinicopathological criteria. In several reports, the distinction between primary intestinal melanoma, metastatic melanoma, and melanoma of unknown primary origin remained uncertain or controversial, particularly in cases with multifocal intestinal involvement or metastatic dissemination. Consequently, the categorization of cases included in this review should be interpreted as a pragmatic approach based on the data available in the publications rather than as an absolute diagnostic classification. In the absence of universally accepted and consistently applied diagnostic criteria, misclassification remains possible (potentially affecting the interpretation of results). Moreover, therapeutic approaches are highly variable and often individualized, underscoring the lack of standardized treatment guidelines, while outcome data are frequently incomplete, retrospective, or limited to short follow-up periods.

From a methodological point of view, the restriction to English-language publications and the inclusion of only studies with available full text might have resulted in the exclusion of relevant data. Moreover, the relatively small number of included studies reflects both the strict selection criteria and the scarcity of robust evidence in this field. Finally, the narrative design of this review, even though appropriate given the heterogeneity of the data, does not allow for quantitative synthesis or meta-analysis.

These limitations highlight the need for larger, multicenter studies, standardized diagnostic criteria, and more consistent reporting practices to improve the current understanding and management of intestinal malignant melanoma. The foundation of an international registry for intestinal malignant melanoma may represent a valuable step toward improving the current understanding of this rare entity. By systematically collecting and standardizing data related to clinical presentation, diagnostic approaches, treatment strategies, and outcomes, such a registry would facilitate more robust analyses and reduce the limitations associated with isolated case reports. Furthermore, fostering collaboration and discussion within the academic community could contribute to the development of more consistent diagnostic criteria and evidence-based management strategies.

## 5. Conclusions

In conclusion, primary small bowel melanoma represents an extremely rare disease that often presents as a solitary lesion in patients without any prior history of cutaneous or mucosal melanoma. However, a proportion of cases classified as primary in the literature may in fact represent melanoma of unknown primary origin, underscoring the diagnostic overlap between entities. The diagnosis is based on excluding any other primary sites through extensive clinical, imaging and endoscopic evaluations. On the other hand, metastatic intestinal melanomas are more common, usually multifocal, and appear in patients with a recorded history of melanoma. It commonly presents with anemia, gastrointestinal bleeding, or intestinal obstruction. However, in both forms, surgery (involving resection) remains the cornerstone of treatment for achieving symptom control, while systemic treatment is typically necessary in cases of metastatic disease. Early diagnosis with advanced endoscopic techniques, like capsule endoscopy and balloon-assisted enteroscopy, is vital to improve outcomes. Despite aggressive multidisciplinary therapies, the prognosis of this disease remains poor for metastatic cases. In contrast, primary intestinal melanoma may be associated with a relatively better prognosis if complete resection is achieved.

The ideal diagnostic approach for suspected intestinal melanoma consists of a stepwise and multimodal strategy. Initial evaluation must include a complete clinical history with focus on prior cutaneous, ocular, or mucosal melanoma, combined with laboratory assessment in cases of anemia or gastrointestinal bleeding. First-line imaging with contrast-enhanced CT or PET-CT is essential for detecting focal lesions, multifocal disease, or even extraintestinal spread. In cases of obscure gastrointestinal bleeding or inconclusive imaging findings, capsule endoscopy is a key non-invasive diagnostic method for identifying small bowel lesions. If a lesion is found or strongly suspected, balloon-assisted enteroscopy allows for direct visualization and biopsy, providing histopathological confirmation. Final diagnosis is based on histopathology supported by immunohistochemistry, including markers like S100, SOX10, HMB-45, and Melan-A. This sequential approach improves diagnostic accuracy and facilitates early therapeutic planning in both primary and metastatic intestinal melanoma.

## Figures and Tables

**Table 1:** Comparative characteristics of primary-like (and MUP-spectrum) vs. metastatic intestinal melanoma, as reported in the analyzed articles. Grouping reflects clinical presentation patterns reported in the literature rather than strict diagnostic categories.

Characteristic	Primary-Like/MUP-Spectrum Intestinal Melanoma	Metastatic Intestinal Melanoma
Frequency	Extremely rare [11]	More common than primary intestinal melanoma [13,19]
History of cutaneous melanoma	Absent [8,10,12]	Usually present, sometimes remote [16,20,22]
Age at presentation	Middle-aged to elderly adults [7,10]	Middle-aged to elderly adults [17,21]
Common location	Duodenum, jejunum, ileum [10,13]	Predominantly jejunum and ileum [16,18]
Clinical presentation	Anemia, obscure GI bleeding, abdominal pain, obstruction, intussusception [11,12,14]	Anemia, melena, obstruction, intussusception, late bleeding [21,22]
Number of lesions	Usually solitary [5,8]	Frequently multiple [17,18]
Endoscopic appearance	Ulcerated or polypoid mass, often amelanotic [5,7]	Pigmented or ulcerated nodules/masses, often multiple [16,17]
Imaging findings	Focal bowel wall thickening or mass ± intussusception [10,12]	Multiple hypermetabolic lesions on CT/PET [16,18]
Histopathology	Melanoma, frequently amelanotic [5,9]	Melanoma, often pigmented [20,21]
Immunohistochemistry	S100, HMB-45, Melan-A positive [8,9]	S100, HMB-45, Melan-A, SOX-10 positive [17,20]
Extraintestinal disease at diagnosis	Absent by definition [8,10]	Frequently present or subsequently identified [18,19]
Diagnostic criteria	Diagnosis of exclusion (Sachs criteria) [5]	Supported by known or newly detected primary melanoma [16,19]
Treatment approach	Surgical resection ± adjuvant therapy [10]	Surgery for complications + systemic oncologic therapy [15,17]
Prognosis	Potentially better if completely resected [8,10]	Generally poor due to disseminated disease [18,22]

## Data Availability

No new data were created or analyzed in this study.
